# (*N*-Benzoyl-*N*-phenyl­hydroxy­laminato)carbon­yl(tri­phenyl­arsine)rhodium(I)

**DOI:** 10.1107/S2414314623003553

**Published:** 2023-04-25

**Authors:** Mokete A. Motente, Johan Venter, Alice Brink

**Affiliations:** a Department of Chemistry, University of the Free State, Bloemfontein, 9301, South Africa; Vienna University of Technology, Austria

**Keywords:** crystal structure, rhodium, *N*-benzoyl-*N*-phenyl­hydroxamic acid, tri­phenyl­arsine

## Abstract

The mol­ecule of the title compound, [Rh(C_13_H_10_NO_2_){As(C_6_H_5_)_3_}(CO)] or [Rh(BPHA)(AsPh_3_)(CO)] (BPHA is the *N*-benzoyl-*N*-phenyl­hydroxy­laminate anion), comprises a bidentate *N*-benzoyl-*N*-phenyl­hydroxy­laminate anion coordinating through the O atoms to the soft Lewis acid, rhodium(I), and two monodentate ligands, *viz*. tri­phenyl­arsine and carbonyl. The resulting CO_2_As coordination environment around the central Rh^I^ atom is distorted square planar.=

## Abstract 2

The mol­ecular structure of the title compound, [Rh(BPHA)(CO)(AsPh_3_)] (where BPHA = *N*-benzoyl-*N*-phenyl­hydroxy­laminate), has a distorted square-planar coordination environment around the central Rh^I^ atom, defined by a CO_2_As coordination set.

## Graphical abstract

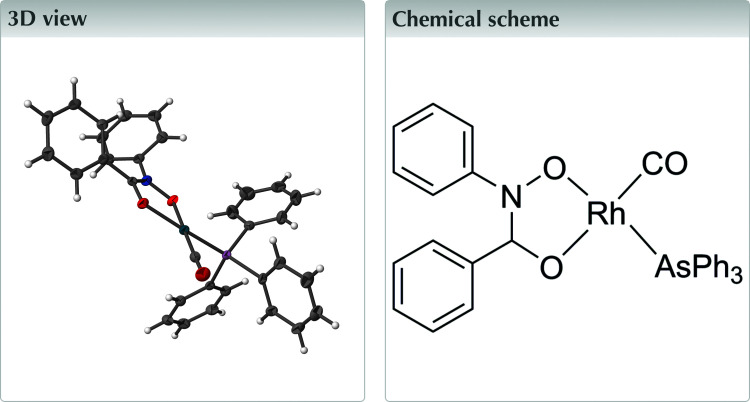

## Structure description

**Figure 1:**
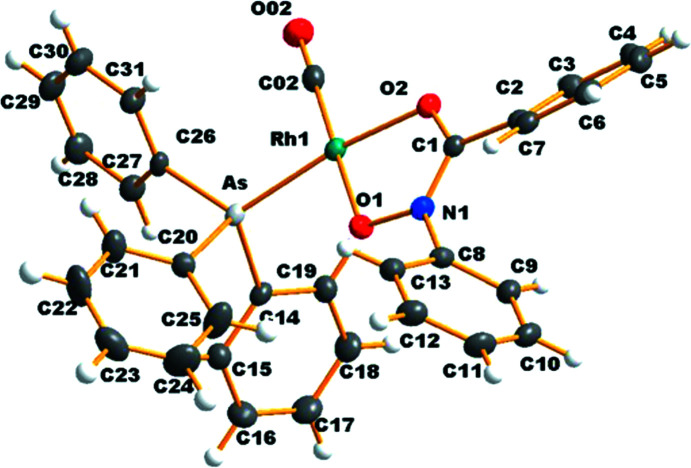
The mol­ecular structure of the title compound, showing atoms with displacement ellipsoids at the 50% probability level.

The title complex, [Rh(BPHA)(AsPh_3_)(CO)], is composed of an *O*,*O*-bidentate *N*-benzoyl-*N*-phenyl­hydroxy­laminate anion, a carbonyl ligand and a monodentate tri­phenyl­arsine ligand, all coordinating to the soft rhodium(I) metal atom (Fig. 1 ▸ and Table 1 ▸). The crystal structure is isotypic with that of [Rh(BPHA)(PPh_3_)(CO)] and shows similar Rh—O and Rh—C bond lengths (2.037/2.089 and 1.809 Å, respectively; Leipoldt & Grobler, 1982 ▸). The coordination environment in the mol­ecule of [Rh(BPHA)(AsPh_3_)(CO)] is distorted square planar, as shown by the small O1—Rh—O2 bite angle of 79.53 (7)°, which is similar to the bite angles of related structures with *O*,*O*-binding five-membered chelate rings reported in the literature (Elmakki *et al.*, 2017 ▸). The C02—Rh—O2 and C02—Rh—O1 angles involving the C02≡O02 carbonyl ligand were also found to deviate from ideal values, at 99.31 (9) and 178.39 (10)°, respectively, similar to those of related structures (Elmakki *et al.*, 2016 ▸).

**Figure 2:**
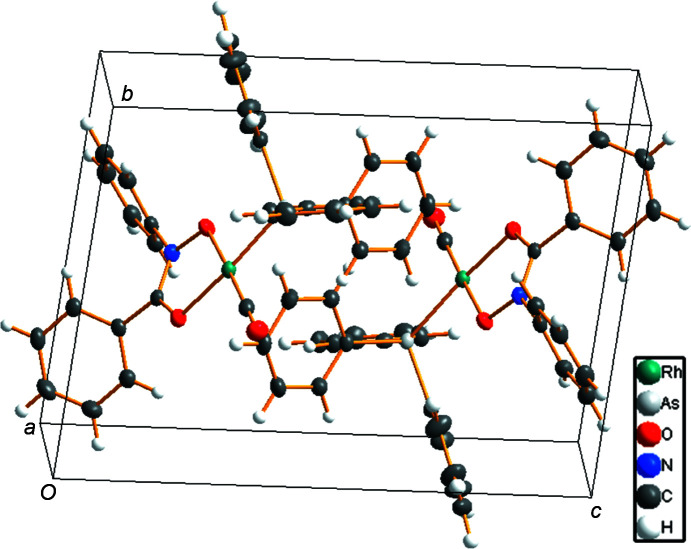
An illustration of the mol­ecular packing in the unit cell of [Rh(BPHA)(CO)(AsPh_3_)], viewed approximately along the *a* axis; atom labels have been omitted for clarity.

The crystal packing is dominated by van der Waals inter­actions (Fig. 2 ▸).

## Synthesis and crystallization

A stepwise process was pursued in the complexation of the rhodium metal atom by the bidentate *N*-phenyl-*N*-benzoyl­hydroxylaminate anion. First, [RhCl(CO)_2_]_2_ was prepared *in situ* by heating RhCl_3_·3H_2_O in 5 ml of di­methyl­formamide under reflux for 30 min, followed by addition of the bidentate ligand to the reaction mixture, which then resulted in the formation of a dicarbonylrhodium species, [Rh(BPHA)(CO)_2_] (Leipoldt & Grobler, 1982 ▸). Rh(BPHA)(CO)_2_] (65 mg) was then dissolved in 5 ml of acetone. Tri­phenyl­arsine (AsPh_3_; 70 mg) was added to the reaction mixture under stirring, resulting in the immediate evolution of CO gas. The reaction mixture was then left to crystallize, resulting in the formation of yellow crystals suitable for X-ray analysis.

## Refinement

Crystal data, data collection and structure refinement details are summarized in Table 2 ▸.

## Supplementary Material

Crystal structure: contains datablock(s) global, I. DOI: 10.1107/S2414314623003553/wm4184sup1.cif

CCDC reference: 2257230

Additional supporting information:  crystallographic information; 3D view; checkCIF report

## Figures and Tables

**Table 1:** Selected bond lengths (Å)

Rh1—As	2.3337 (4)	Rh1—O1	2.0338 (18)
Rh1—O2	2.0682 (17)	Rh1—C02	1.813 (3)

**Table 2:** Experimental details

Crystal data	
Chemical formula	[Rh(C_13_H_10_NO_2_)(C_18_H_15_As)(CO)]
*M*_r_	649.36
Crystal system, space group	Triclinic, *P* 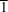
Temperature (K)	100
*a*, *b*, *c* (Å)	9.5178 (17), 10.1995 (19), 14.589 (2)
α, β, γ (°)	81.516 (6), 83.142 (6), 72.351 (7)
*V* (Å^3^)	1330.7 (4)
*Z*	2
Radiation type	Mo *K*α
μ (mm^−1^)	1.91
Crystal size (mm)	0.21 × 0.13 × 0.03

Data collection	
Diffractometer	Bruker APEXII CCD
Absorption correction	Multi-scan (*SADABS*; Krause *et al.*, 2015 ▸)
*T*_min_, *T*_max_	0.634, 0.746
No. of measured, independent and observed [*I* > 2σ(*I*)] reflections	38485, 6425, 5747
*R*_int_	0.065
(sin θ/λ)_max_ (Å^−1^)	0.660

Refinement	
*R*[*F* ^2^ > 2σ(*F* ^2^)], *wR*(*F* ^2^), *S*	0.030, 0.074, 1.07
No. of reflections	6425
No. of parameters	343
H-atom treatment	H-atom parameters constrained
Δρ_max_, Δρ_min_ (e Å^−3^)	1.14, −0.61

Computer programs: *APEX2* and *SAINT* (Bruker, 2012 ▸), *SHELXS* (Sheldrick, 2008 ▸), *SHELXL* (Sheldrick, 2015 ▸), *OLEX2* (Dolomanov *et al.*, 2009 ▸) and *DIAMOND* (Brandenburg & Putz, 2005 ▸).

## full crystallographic data

### Crystal data

**Table:**

[Rh(C_13_H_10_NO_2_)(C_18_H_15_As)(CO)]	*Z* = 2
*M**_r_* = 649.36	*F*(000) = 652
Triclinic, *P*1	*D*_x_ = 1.621 Mg m^−^^3^
*a* = 9.5178 (17) Å	Mo *K*α radiation, λ = 0.71073 Å
*b* = 10.1995 (19) Å	Cell parameters from 9339 reflections
*c* = 14.589 (2) Å	θ = 2.3–28.3°
α = 81.516 (6)°	µ = 1.91 mm^−^^1^
β = 83.142 (6)°	*T* = 100 K
γ = 72.351 (7)°	Plate, yellow
*V* = 1330.7 (4) Å^3^	0.21 × 0.13 × 0.03 mm

### Data collection

**Table:**

Bruker APEXII CCD diffractometer	5747 reflections with *I* > 2σ(*I*)
φ and ω scans	*R*_int_ = 0.065
Absorption correction: multi-scan (SADABS; Krause *et al.*, 2015)	θ_max_ = 28.0°, θ_min_ = 2.1°
*T*_min_ = 0.634, *T*_max_ = 0.746	*h* = −12→12
38485 measured reflections	*k* = −13→13
6425 independent reflections	*l* = −19→19

### Refinement

**Table:**

Refinement on *F*^2^	0 restraints
Least-squares matrix: full	Hydrogen site location: inferred from neighbouring sites
*R*[*F*^2^ > 2σ(*F*^2^)] = 0.030	H-atom parameters constrained
*wR*(*F*^2^) = 0.074	*w* = 1/[σ^2^(*F*_o_^2^) + (0.0141*P*)^2^ + 1.8613*P*] where *P* = (*F*_o_^2^ + 2*F*_c_^2^)/3
*S* = 1.07	(Δ/σ)_max_ = 0.002
6425 reflections	Δρ_max_ = 1.14 e Å^−^^3^
343 parameters	Δρ_min_ = −0.61 e Å^−^^3^

### Special details

**Table:**

Geometry. All esds (except the esd in the dihedral angle between two l.s. planes) are estimated using the full covariance matrix. The cell esds are taken into account individually in the estimation of esds in distances, angles and torsion angles; correlations between esds in cell parameters are only used when they are defined by crystal symmetry. An approximate (isotropic) treatment of cell esds is used for estimating esds involving l.s. planes.

### Fractional atomic coordinates and isotropic or equivalent isotropic displacement parameters (Å^2^)

**Table:**

	*x*	*y*	*z*	*U*_iso_*/*U*_eq_	
Rh1	0.25715 (2)	0.53736 (2)	0.27266 (2)	0.01779 (6)	
As	0.12957 (3)	0.72691 (2)	0.35181 (2)	0.01699 (6)	
O2	0.39711 (19)	0.38301 (18)	0.19949 (12)	0.0210 (4)	
O1	0.42339 (19)	0.62308 (18)	0.22612 (13)	0.0217 (4)	
O02	0.0140 (2)	0.4121 (2)	0.33366 (15)	0.0313 (4)	
N1	0.5303 (2)	0.5365 (2)	0.17371 (15)	0.0207 (4)	
C1	0.5119 (3)	0.4183 (2)	0.15914 (16)	0.0169 (4)	
C26	−0.0798 (3)	0.7588 (2)	0.38511 (18)	0.0197 (5)	
C8	0.6478 (3)	0.5925 (3)	0.13234 (17)	0.0198 (5)	
C14	0.2129 (3)	0.7244 (3)	0.46672 (17)	0.0189 (5)	
C20	0.1411 (3)	0.9043 (3)	0.28753 (17)	0.0207 (5)	
C15	0.2025 (3)	0.8463 (3)	0.50321 (19)	0.0241 (5)	
H15	0.1470	0.9331	0.4741	0.029*	
C7	0.6819 (3)	0.3597 (3)	0.01447 (18)	0.0212 (5)	
H7	0.6631	0.4551	−0.0082	0.025*	
C27	−0.1418 (3)	0.7798 (3)	0.47479 (19)	0.0237 (5)	
H27	−0.0816	0.7815	0.5218	0.028*	
C13	0.6118 (3)	0.7151 (3)	0.07297 (19)	0.0241 (5)	
H13	0.5113	0.7637	0.0637	0.029*	
C2	0.6182 (3)	0.3193 (3)	0.10122 (17)	0.0190 (5)	
C19	0.2907 (3)	0.5971 (3)	0.51116 (18)	0.0237 (5)	
H19	0.2958	0.5138	0.4873	0.028*	
C02	0.1092 (3)	0.4594 (3)	0.31095 (18)	0.0224 (5)	
C6	0.7729 (3)	0.2591 (3)	−0.03845 (19)	0.0250 (5)	
H6	0.8153	0.2862	−0.0978	0.030*	
C28	−0.2931 (3)	0.7983 (3)	0.4956 (2)	0.0304 (6)	
H28	−0.3356	0.8119	0.5570	0.036*	
C9	0.7931 (3)	0.5246 (3)	0.15114 (19)	0.0232 (5)	
H9	0.8160	0.4439	0.1950	0.028*	
C3	0.6475 (3)	0.1796 (3)	0.13401 (19)	0.0247 (5)	
H3	0.6039	0.1518	0.1928	0.030*	
C18	0.3607 (3)	0.5920 (3)	0.59044 (19)	0.0280 (6)	
H18	0.4137	0.5054	0.6211	0.034*	
C11	0.8703 (3)	0.6955 (3)	0.0429 (2)	0.0296 (6)	
H11	0.9472	0.7295	0.0106	0.036*	
C31	−0.1692 (3)	0.7556 (3)	0.3171 (2)	0.0268 (6)	
H31	−0.1268	0.7393	0.2559	0.032*	
C12	0.7241 (3)	0.7658 (3)	0.0274 (2)	0.0290 (6)	
H12	0.7011	0.8487	−0.0144	0.035*	
C16	0.2733 (3)	0.8406 (3)	0.58217 (19)	0.0284 (6)	
H16	0.2669	0.9234	0.6069	0.034*	
C4	0.7403 (3)	0.0805 (3)	0.0812 (2)	0.0298 (6)	
H4	0.7616	−0.0149	0.1044	0.036*	
C10	0.9049 (3)	0.5768 (3)	0.1046 (2)	0.0275 (6)	
H10	1.0053	0.5304	0.1155	0.033*	
C17	0.3531 (3)	0.7141 (3)	0.6246 (2)	0.0301 (6)	
H17	0.4033	0.7106	0.6779	0.036*	
C5	0.8022 (3)	0.1204 (3)	−0.0057 (2)	0.0300 (6)	
H5	0.8644	0.0523	−0.0423	0.036*	
C29	−0.3810 (3)	0.7971 (3)	0.4275 (2)	0.0332 (6)	
H29	−0.4841	0.8107	0.4420	0.040*	
C25	0.2759 (3)	0.9299 (3)	0.2743 (2)	0.0390 (7)	
H25	0.3614	0.8592	0.2938	0.047*	
C24	0.2892 (4)	1.0577 (4)	0.2327 (2)	0.0417 (8)	
H24	0.3829	1.0747	0.2256	0.050*	
C21	0.0196 (3)	1.0048 (3)	0.2526 (2)	0.0324 (6)	
H21	−0.0736	0.9871	0.2583	0.039*	
C23	0.1672 (4)	1.1593 (3)	0.2019 (2)	0.0368 (7)	
H23	0.1750	1.2480	0.1759	0.044*	
C30	−0.3194 (3)	0.7760 (3)	0.3380 (2)	0.0340 (7)	
H30	−0.3802	0.7755	0.2911	0.041*	
C22	0.0335 (4)	1.1316 (3)	0.2091 (3)	0.0442 (8)	
H22	−0.0499	1.1994	0.1841	0.053*	

### Atomic displacement parameters (Å^2^)

**Table:**

	*U*^11^	*U*^22^	*U*^33^	*U*^12^	*U*^13^	*U*^23^
Rh1	0.01456 (9)	0.02186 (10)	0.01630 (10)	−0.00485 (7)	0.00038 (7)	−0.00246 (7)
As	0.01309 (12)	0.02045 (12)	0.01611 (13)	−0.00335 (9)	−0.00019 (9)	−0.00199 (9)
O2	0.0163 (8)	0.0257 (9)	0.0210 (9)	−0.0066 (7)	0.0018 (7)	−0.0040 (7)
O1	0.0176 (8)	0.0245 (9)	0.0235 (10)	−0.0066 (7)	0.0048 (7)	−0.0087 (7)
O02	0.0258 (10)	0.0340 (10)	0.0364 (12)	−0.0152 (8)	0.0031 (8)	−0.0022 (9)
N1	0.0172 (10)	0.0252 (10)	0.0200 (11)	−0.0067 (8)	0.0012 (8)	−0.0046 (8)
C1	0.0154 (11)	0.0209 (11)	0.0138 (11)	−0.0045 (9)	−0.0014 (8)	−0.0012 (8)
C26	0.0126 (10)	0.0187 (11)	0.0253 (13)	−0.0023 (8)	0.0003 (9)	−0.0014 (9)
C8	0.0196 (11)	0.0245 (12)	0.0178 (12)	−0.0091 (9)	0.0011 (9)	−0.0066 (9)
C14	0.0139 (11)	0.0261 (12)	0.0159 (12)	−0.0045 (9)	−0.0007 (9)	−0.0031 (9)
C20	0.0212 (12)	0.0222 (11)	0.0182 (12)	−0.0065 (9)	0.0007 (9)	−0.0019 (9)
C15	0.0234 (13)	0.0254 (12)	0.0220 (13)	−0.0053 (10)	−0.0005 (10)	−0.0033 (10)
C7	0.0176 (11)	0.0281 (12)	0.0190 (12)	−0.0074 (10)	−0.0039 (9)	−0.0023 (9)
C27	0.0212 (12)	0.0280 (13)	0.0215 (13)	−0.0086 (10)	0.0014 (10)	−0.0014 (10)
C13	0.0223 (12)	0.0258 (12)	0.0260 (14)	−0.0085 (10)	−0.0019 (10)	−0.0056 (10)
C2	0.0144 (11)	0.0244 (12)	0.0191 (12)	−0.0053 (9)	−0.0025 (9)	−0.0056 (9)
C19	0.0224 (12)	0.0245 (12)	0.0218 (13)	−0.0039 (10)	−0.0005 (10)	−0.0024 (10)
C02	0.0218 (12)	0.0228 (12)	0.0191 (13)	−0.0018 (10)	−0.0015 (10)	−0.0019 (9)
C6	0.0183 (12)	0.0381 (14)	0.0191 (13)	−0.0076 (11)	−0.0006 (10)	−0.0074 (10)
C28	0.0236 (13)	0.0376 (15)	0.0277 (15)	−0.0089 (11)	0.0093 (11)	−0.0058 (12)
C9	0.0202 (12)	0.0259 (12)	0.0235 (13)	−0.0059 (10)	−0.0029 (10)	−0.0042 (10)
C3	0.0257 (13)	0.0275 (13)	0.0218 (13)	−0.0086 (10)	−0.0020 (10)	−0.0038 (10)
C18	0.0286 (14)	0.0314 (13)	0.0204 (14)	−0.0045 (11)	−0.0044 (11)	0.0010 (10)
C11	0.0248 (13)	0.0402 (15)	0.0294 (15)	−0.0187 (12)	0.0039 (11)	−0.0069 (12)
C31	0.0197 (12)	0.0335 (14)	0.0267 (14)	−0.0051 (10)	−0.0008 (10)	−0.0087 (11)
C12	0.0314 (14)	0.0333 (14)	0.0258 (15)	−0.0154 (12)	−0.0009 (11)	−0.0023 (11)
C16	0.0336 (15)	0.0320 (14)	0.0210 (14)	−0.0097 (12)	−0.0012 (11)	−0.0083 (11)
C4	0.0307 (14)	0.0233 (12)	0.0339 (16)	−0.0036 (11)	−0.0030 (12)	−0.0074 (11)
C10	0.0185 (12)	0.0381 (15)	0.0286 (15)	−0.0095 (11)	0.0004 (10)	−0.0115 (11)
C17	0.0311 (15)	0.0407 (16)	0.0190 (14)	−0.0101 (12)	−0.0046 (11)	−0.0040 (11)
C5	0.0222 (13)	0.0328 (14)	0.0337 (16)	−0.0007 (11)	−0.0025 (11)	−0.0153 (12)
C29	0.0153 (12)	0.0405 (16)	0.0431 (18)	−0.0075 (11)	0.0029 (12)	−0.0081 (13)
C25	0.0228 (14)	0.0416 (17)	0.048 (2)	−0.0094 (12)	−0.0029 (13)	0.0112 (14)
C24	0.0386 (17)	0.0452 (18)	0.046 (2)	−0.0252 (15)	0.0003 (15)	0.0052 (15)
C21	0.0230 (13)	0.0269 (13)	0.0418 (18)	−0.0032 (11)	0.0029 (12)	0.0005 (12)
C23	0.0518 (19)	0.0258 (13)	0.0319 (17)	−0.0152 (13)	0.0118 (14)	−0.0041 (12)
C30	0.0199 (13)	0.0419 (16)	0.0415 (18)	−0.0075 (12)	−0.0050 (12)	−0.0101 (13)
C22	0.0405 (18)	0.0228 (14)	0.058 (2)	0.0006 (13)	0.0035 (16)	0.0053 (14)

### Geometric parameters (Å, º)

**Table:**

Rh1—As	2.3337 (4)	C6—C5	1.379 (4)
Rh1—O2	2.0682 (17)	C28—H28	0.9500
Rh1—O1	2.0338 (18)	C28—C29	1.376 (4)
Rh1—C02	1.813 (3)	C9—H9	0.9500
As—C26	1.933 (2)	C9—C10	1.395 (4)
As—C14	1.932 (2)	C3—H3	0.9500
As—C20	1.939 (2)	C3—C4	1.386 (4)
O2—C1	1.301 (3)	C18—H18	0.9500
O1—N1	1.367 (3)	C18—C17	1.387 (4)
O02—C02	1.144 (3)	C11—H11	0.9500
N1—C1	1.319 (3)	C11—C12	1.387 (4)
N1—C8	1.437 (3)	C11—C10	1.375 (4)
C1—C2	1.479 (3)	C31—H31	0.9500
C26—C27	1.388 (4)	C31—C30	1.384 (4)
C26—C31	1.392 (4)	C12—H12	0.9500
C8—C13	1.387 (4)	C16—H16	0.9500
C8—C9	1.385 (4)	C16—C17	1.381 (4)
C14—C15	1.395 (3)	C4—H4	0.9500
C14—C19	1.393 (3)	C4—C5	1.391 (4)
C20—C25	1.371 (4)	C10—H10	0.9500
C20—C21	1.384 (4)	C17—H17	0.9500
C15—H15	0.9500	C5—H5	0.9500
C15—C16	1.388 (4)	C29—H29	0.9500
C7—H7	0.9500	C29—C30	1.385 (4)
C7—C2	1.397 (4)	C25—H25	0.9500
C7—C6	1.391 (4)	C25—C24	1.390 (4)
C27—H27	0.9500	C24—H24	0.9500
C27—C28	1.395 (4)	C24—C23	1.371 (5)
C13—H13	0.9500	C21—H21	0.9500
C13—C12	1.385 (4)	C21—C22	1.391 (4)
C2—C3	1.389 (4)	C23—H23	0.9500
C19—H19	0.9500	C23—C22	1.374 (5)
C19—C18	1.390 (4)	C30—H30	0.9500
C6—H6	0.9500	C22—H22	0.9500
			
O2—Rh1—As	171.06 (5)	C29—C28—H28	119.8
O1—Rh1—As	91.66 (5)	C8—C9—H9	120.6
O1—Rh1—O2	79.53 (7)	C8—C9—C10	118.8 (2)
C02—Rh1—As	89.53 (8)	C10—C9—H9	120.6
C02—Rh1—O2	99.31 (9)	C2—C3—H3	119.9
C02—Rh1—O1	178.39 (10)	C4—C3—C2	120.2 (3)
C26—As—Rh1	118.78 (7)	C4—C3—H3	119.9
C26—As—C20	103.15 (10)	C19—C18—H18	120.1
C14—As—Rh1	112.68 (7)	C17—C18—C19	119.7 (3)
C14—As—C26	104.81 (11)	C17—C18—H18	120.1
C14—As—C20	100.97 (11)	C12—C11—H11	119.7
C20—As—Rh1	114.44 (8)	C10—C11—H11	119.7
C1—O2—Rh1	111.54 (15)	C10—C11—C12	120.6 (3)
N1—O1—Rh1	110.33 (13)	C26—C31—H31	119.9
O1—N1—C8	114.38 (19)	C30—C31—C26	120.3 (3)
C1—N1—O1	119.1 (2)	C30—C31—H31	119.9
C1—N1—C8	126.2 (2)	C13—C12—C11	119.9 (3)
O2—C1—N1	119.3 (2)	C13—C12—H12	120.0
O2—C1—C2	117.1 (2)	C11—C12—H12	120.0
N1—C1—C2	123.5 (2)	C15—C16—H16	120.1
C27—C26—As	122.12 (19)	C17—C16—C15	119.8 (3)
C27—C26—C31	119.6 (2)	C17—C16—H16	120.1
C31—C26—As	118.27 (19)	C3—C4—H4	119.9
C13—C8—N1	118.2 (2)	C3—C4—C5	120.2 (3)
C9—C8—N1	120.5 (2)	C5—C4—H4	119.9
C9—C8—C13	121.3 (2)	C9—C10—H10	119.9
C15—C14—As	121.74 (19)	C11—C10—C9	120.1 (3)
C19—C14—As	118.28 (19)	C11—C10—H10	119.9
C19—C14—C15	119.9 (2)	C18—C17—H17	119.6
C25—C20—As	118.6 (2)	C16—C17—C18	120.7 (3)
C25—C20—C21	118.7 (3)	C16—C17—H17	119.6
C21—C20—As	122.7 (2)	C6—C5—C4	119.7 (2)
C14—C15—H15	120.1	C6—C5—H5	120.1
C16—C15—C14	119.9 (2)	C4—C5—H5	120.1
C16—C15—H15	120.1	C28—C29—H29	120.0
C2—C7—H7	120.2	C28—C29—C30	120.1 (3)
C6—C7—H7	120.2	C30—C29—H29	120.0
C6—C7—C2	119.5 (2)	C20—C25—H25	119.5
C26—C27—H27	120.2	C20—C25—C24	121.0 (3)
C26—C27—C28	119.7 (3)	C24—C25—H25	119.5
C28—C27—H27	120.2	C25—C24—H24	120.0
C8—C13—H13	120.4	C23—C24—C25	120.0 (3)
C12—C13—C8	119.1 (3)	C23—C24—H24	120.0
C12—C13—H13	120.4	C20—C21—H21	119.9
C7—C2—C1	123.2 (2)	C20—C21—C22	120.3 (3)
C3—C2—C1	117.0 (2)	C22—C21—H21	119.9
C3—C2—C7	119.7 (2)	C24—C23—H23	120.3
C14—C19—H19	120.1	C24—C23—C22	119.5 (3)
C18—C19—C14	119.8 (2)	C22—C23—H23	120.3
C18—C19—H19	120.1	C31—C30—C29	120.0 (3)
O02—C02—Rh1	178.5 (2)	C31—C30—H30	120.0
C7—C6—H6	119.7	C29—C30—H30	120.0
C5—C6—C7	120.7 (3)	C21—C22—H22	119.8
C5—C6—H6	119.7	C23—C22—C21	120.3 (3)
C27—C28—H28	119.8	C23—C22—H22	119.8
C29—C28—C27	120.4 (3)		
			
Rh1—O2—C1—N1	4.4 (3)	C8—C9—C10—C11	1.5 (4)
Rh1—O2—C1—C2	−177.62 (16)	C14—C15—C16—C17	−0.4 (4)
Rh1—O1—N1—C1	1.5 (3)	C14—C19—C18—C17	−0.2 (4)
Rh1—O1—N1—C8	175.94 (16)	C20—C25—C24—C23	−1.8 (6)
As—C26—C27—C28	−178.1 (2)	C20—C21—C22—C23	−1.2 (5)
As—C26—C31—C30	179.1 (2)	C15—C14—C19—C18	−1.6 (4)
As—C14—C15—C16	−175.2 (2)	C15—C16—C17—C18	−1.4 (4)
As—C14—C19—C18	175.6 (2)	C7—C2—C3—C4	0.4 (4)
As—C20—C25—C24	−176.8 (3)	C7—C6—C5—C4	−0.1 (4)
As—C20—C21—C22	178.4 (3)	C27—C26—C31—C30	1.4 (4)
O2—C1—C2—C7	137.2 (2)	C27—C28—C29—C30	0.6 (5)
O2—C1—C2—C3	−39.0 (3)	C13—C8—C9—C10	−4.1 (4)
O1—N1—C1—O2	−4.1 (3)	C2—C7—C6—C5	−0.8 (4)
O1—N1—C1—C2	178.1 (2)	C2—C3—C4—C5	−1.3 (4)
O1—N1—C8—C13	−58.9 (3)	C19—C14—C15—C16	1.9 (4)
O1—N1—C8—C9	121.5 (2)	C19—C18—C17—C16	1.7 (4)
N1—C1—C2—C7	−45.0 (4)	C6—C7—C2—C1	−175.5 (2)
N1—C1—C2—C3	138.8 (3)	C6—C7—C2—C3	0.6 (4)
N1—C8—C13—C12	−175.6 (2)	C28—C29—C30—C31	0.3 (5)
N1—C8—C9—C10	175.5 (2)	C9—C8—C13—C12	4.0 (4)
C1—N1—C8—C13	115.1 (3)	C3—C4—C5—C6	1.1 (4)
C1—N1—C8—C9	−64.5 (3)	C31—C26—C27—C28	−0.5 (4)
C1—C2—C3—C4	176.7 (2)	C12—C11—C10—C9	1.2 (4)
C26—C27—C28—C29	−0.5 (4)	C10—C11—C12—C13	−1.3 (4)
C26—C31—C30—C29	−1.3 (5)	C25—C20—C21—C22	−3.2 (5)
C8—N1—C1—O2	−177.9 (2)	C25—C24—C23—C22	−2.7 (5)
C8—N1—C1—C2	4.3 (4)	C24—C23—C22—C21	4.2 (5)
C8—C13—C12—C11	−1.3 (4)	C21—C20—C25—C24	4.7 (5)
